# Hemocompatible gelatin-glycidyl methacrylate/graphene oxide composite hydrogels for vascular catheter applications

**DOI:** 10.1038/s41598-025-93040-2

**Published:** 2025-03-25

**Authors:** Asmaa Kh. Atef, Tahia B. Mostafa, Hazem M. El-Sherif

**Affiliations:** 1 Chemistry Department, Faculty of Women for Art, Science and Education, Ain Shams University, Héliopolis, Cairo, Egypt; 2 Polymers and Pigments Department, National Research Centre, Cairo, Egypt

**Keywords:** Gelatin, Graphene oxide, Hydrogels, Blood contacting biomaterials, Vascular catheters, Thrombogenicity, Amino sugars, Chemical modification, Graphene

## Abstract

The development of biocompatible and hemocompatible materials is crucial for various biomedical applications. In this study, gelatin (Gel) was modified using glycidyl methacrylate (GMA) to create a photo-curable macromer (Gel-GMA), facilitating subsequent crosslinking via UV radiation. Additionally, a composite was prepared by incorporating graphene oxide (GO) into the modified gelatin matrix (Gel-GMA/GO). Structural and morphological analyses revealed macroporous or interconnected structures in the hydrogels and composites, resulting in high swelling capacities (> 1050%). Hemolysis testing demonstrated minimal hemolytic activity for both Gel-GMA and Gel-GMA/GO hydrogels, confirming their excellent hemocompatibility (0.54 and 0.50% respectively). Prothrombin time (PT) tests indicated negligible differences compared to normal blood, suggesting low thrombogenicity. The incorporation of GO reduced the PT to 12.9s. Furthermore, in vitro degradation studies under simulated blood conditions revealed moderate degradation rates) for Gel-GMA and Gel-GMA/GO hydrogels (37 and 18%, respectively) after 30 days. Viability assays on MRC-5 cells exposed to composite extracts up to 500 µg/ml showed consistent cell viability (more than 91.7%), with a slight reduction at higher concentrations. These findings underscore the potential of the hydrogels for applications such as vascular catheters, highlighting their biocompatibility, hemocompatibility, and controlled degradability.

## Introduction

Blood-contacting medical devices are widely and practically used, and their use is expected to grow significantly as lifespans increase and technology advances^1–4^. The Centres for Disease Control and Prevention report that 15–25% of hospitalized patients undergo catheterization, a number that is steadily climbing^5^. Despite their benefits, these devices carry a substantial risk of thrombosis, which remains a leading cause of morbidity and mortality associated with their use.

Addressing this issue necessitates a multidisciplinary approach that includes the development of more effective and safer antithrombotic agents, as well as the creation of less thrombogenic biomaterials. Until these advancements are realized, clinicians must carefully balance the prevention of thrombosis with the risk of inducing severe bleeding^6–8^. Any device, regardless of its type or duration of use, exposes blood to foreign surfaces, potentially triggering immunological responses or coagulation and leading to clot formation. When blood contacts a catheter, blood cells and proteins accumulate on its surface, a process influenced significantly by the catheter’s material and design^9–11^. Catheter-related thrombi can cause a range of severe complications, such as pulmonary embolism, infections, loss of venous access, deep vein thrombosis, extended hospital stays, increased patient morbidity, and catheter malfunction.

Currently, catheters used for vascular access are typically made from polyurethanes or silicones, a design that has remained largely unchanged for decades. While these materials offer strength and flexibility, they also tend to be thrombogenic. Their hydrophobic nature results in a lack of lubricity, causing significant friction between the biomedical materials and biological tissues^12,13^. Various coatings and surface treatments have been developed to address this issue, but many of these coatings lack durability, and thrombus formation can still occur, especially in patients who need long-term catheterization^14^. For example, efforts to reduce thrombus formation have included coating the blood-contacting surface of cardiovascular implants by reacting porcine gelatin with increasing amounts of lysine diisocyanate ethyl ester^15^. Ideally, catheters should possess mechanical stability, flexibility, and the ability to absorb toxins^16^.

Despite advances in catheter design, modern catheters frequently fail to meet al.l desired criteria simultaneously. Fortunately, hydrogels, which are emerging biomedical materials known for their softness and customizable shapes, show significant potential in addressing these challenges. Hydrogels consist of a three-dimensional (3D) network formed by hydrophilic polymers with physical or chemical crosslinks, making them suitable for catheter coatings due to their excellent hydrophilicity, high water content, porous structures, and smooth surfaces^5^. For instance, research by Yong et al. demonstrated that the lubricating and antifouling properties of catheters were significantly enhanced, with a more than tenfold reduction in the coefficient of friction compared to uncoated substrates^17^. However, many hydrogels have intrinsic issues with swelling and weakening properties, which can make them unsuitable for in vivo use and may result in dangerous fragments and foreign body residues.

Traditional catheters often exhibit poor drainage and absorption properties due to their simple tubular structures and dense, nonporous surfaces^18,19^. A recent study explored a multi-interpenetrating network of polyacrylamide, sodium alginate, and chitosan, which demonstrated both desirable mechanical strength and flexibility compatible with the abdominal wall^16^. Additionally, a heat-treated composite hydrogel of poly(vinyl alcohol) and poly(acrylic acid) was shown to combine the mechanical strength and durability of thermoplastic polyurethanes with a non-thrombogenic surface, without the need for chemical cross-linking agents^20^. To further address these issues, gelatin, a naturally processed biopolymer, was selected for its controlled properties and high physiological compatibility. Gelatin offers greater versatility in medical and pharmaceutical applications than other natural or synthetic polymers and is ideal for various medical uses, including regenerative medicine and minimally invasive surgery^21,22^.Recent advancements in hydrogel materials for vascular catheter applications have emphasized the necessity of hemocompatible, durable, and scalable solutions. Conventional catheter materials, often based on hydrophobic polymers or fragile surface coatings, are prone to degradation, thrombosis, and insufficient mechanical robustness. To address these limitations, we present a novel composite hydrogel system integrating glycidyl methacrylate (GMA)-modified gelatin with graphene oxide (GO).

This innovative combination leverages the natural biocompatibility of gelatin and the hydrophilic, reinforcing properties of GO to produce a hydrogel with superior mechanical integrity, controlled degradability, and enhanced hemocompatibility. The UV-induced crosslinking process employed for fabrication offers a scalable and efficient method to form stable hydrogels with interconnected macroporous structures, ideal for blood-contacting applications. The composite’s unique properties, including high water content and a neutral surface charge, are pivotal in minimizing the adhesion of blood components, thereby reducing thrombosis risk. Additionally, the macroporous architecture promotes efficient fluid absorption and facilitates the diffusion of biological fluids, supporting vascular catheter functionality. The lubricious nature of the hydrogel, enabled by its high-water content, potentially minimizes vessel trauma and inflammation, further establishing its suitability for biomedical applications. By addressing critical challenges in vascular catheter design, this study demonstrates the potential of Gel-GMA/GO hydrogels as next-generation materials for blood-contacting medical devices.

## Materials and methods

### Materials

Gelatin from bovine skin (gel strength ~ 225 g Bloom, Type B, glycidyl-methacrylate (GMA, 97%, contains 100 ppm monomethyl ether hydroquinone as an inhibitor), Irgacure 2959 (2-hydroxy-4′-(2-hydroxyethoxy)-2-methylpropiophenone) as a photoinitiator were purchased from Sigma-Aldrich, USA. Sodium hydroxide, hydrochloric acid, sodium carbonate, sodium bicarbonate, and pure graphite fine powder were purchased from Loba Chemie Ltd., India). 98% Sulphuric acid, 30% hydrogen peroxide, potassium permanganate, potassium nitrate, and phosphoric acid were obtained from BDH Chemicals Ltd. Poole England. 25% Ammonia solution, 99% ethanol, potassium chloride, sodium chloride, disodium phosphate, and monopotassium phosphate were purchased from El-Gomhouria Co. for Drugs and Chemicals, Cairo, Egypt. All chemicals were used without further purification. Double distilled water (DW) was used throughout this work.

### Experimental techniques

#### Synthesis of gelatin-glycidyl methacrylate (Gel-GMA) macromers

A 10% (w/v) gelatin solution was dissolved with stirring in pH 9.4 carbonate-bicarbonate buffer solution at 50 °C. The reaction commenced with the addition of glycidyl methacrylate (GMA) under vigorous stirring at 500 rpm at the same temperature. GMA was added in ten successive portions, each according to the desired ratio (x ml GMA/g Gel), namely, 0.1:1, 0.4:1, 0.7:1, and 1:1, resulting in modified gelatins designated as G1, G2, G3, and G4, respectively. pH was adjusted back to 9.4 after each addition. The reaction proceeded for 180 min and was halted by adjusting the pH to 7.4 using 1 N HCl. The reaction mixture was filtered using standard filter paper and dialyzed with a 14 kDa molecular-weight-cut-off membrane at 50 °C for 36 h against ultrapure water. Water was changed every 4 h to ensure complete removal of low molecular weight gelatin and methacrylic acid reaction by-products. The dialyzed modified gelatin was then frozen, lyophilized, and stored in the dark at 4 °C for further use^23^.

#### Synthesis of Gel-GMA hydrogels

Gelatin-glycidyl methacrylate (Gel-GMA) solutions were prepared by dissolving the desired macromers at a concentration of 10% (w/v) in PBS solution (0.01 M, pH = 7.4) at 50 °C. Subsequently, 0.5% (w/v) Irgacure 2959 was added to initiate the photoreaction, which was carried out using UV light at 365 nm (CL-1000, Funakoshi Co., Ltd., Tokyo, Japan) positioned at a distance of 10 cm for 15 min. Following the curing process, the hydrogels were sliced into disks with a diameter of 10 mm and a thickness of approximately 2 mm^23^.

#### Synthesis of graphene oxide (GO) from graphite powder

GO was synthesized following a modified version of the Hummer’s method^24^. Initially, 1 g of graphite and 9.89 mmole of KNO_3_ were combined with 50 ml of concentrated H_2_SO_4_ and 3 ml of H3PO4, then stirred in an ice bath for 120 min. Subsequently, 37.96 mmole of KMnO_4_ was gradually added while ensuring the mixture temperature remained below 5 °C. The suspension was allowed to react for 2 h in the ice bath before stirring at 40 °C for an additional 60 min. A color change from black to deep green indicated the successful addition of KMnO_4_. The suspension was then transferred to a water bath maintained at 40–50 °C and stirred continuously for approximately 24 h until the color changed to pale brown. Heating the mixture to 98 °C for 30 min, with slow addition of water under stirring, resulted in a dark brown suspension. Finally, 3 ml of H_2_O_2_ (30%) was added dropwise to neutralize excess potassium permanganate. The washing process involved repeated centrifugation at 4000 rpm for 4 h, followed by decanting the supernatant. The pH of the collected material was adjusted to around 7 using a universal indicator. The collected material (GO) was then stirred in distilled water at room temperature for 24 h.

#### Synthesis of Gel-GMA/GO composites

To synthesize Gel-GMA/GO composites, we utilized the procedure outlined in Sect. 2.2.2, incorporating 0.2% GO as specified.

### Characterization of graphene oxide, hydrogels, and composites thereof

#### Swelling behavior of hydrogel

The swelling behavior of hydrogels was assessed by immersing freeze-dried hydrogels in distilled water (DW) and phosphate-buffered solution (PBS) with a pH of 7.4 at 37 °C, simulating blood media conditions. The degree of swelling was determined by measuring the uptake of water into the hydrogels gravimetrically once equilibrium was reached^25^. Prior to weight measurements, the samples were gently blotted with filter paper to remove any surface water. The swelling percentage was calculated using Eq. 1:

1 $${\text{Swelling percentage }} = \left( {\frac{{W_{s} - W_{d} }}{{W_{d} }}} \right) \times 100$$

where W_**s**_ and W_**d**_ correspond to the weight of swollen hydrogel at equilibrium and after lyophilization, respectively.

#### Porosity of hydrogels

Porosity measurements were conducted using the hexane displacement method. Initially, hydrogels were cut into smaller cylindrical pieces of approximately equal dimensions, and their diameter and height were measured using a screw gauge to determine their volumes. These hydrogel pieces were then immersed in a solution of n-hexane for 30 min, allowing the hexane to fill the pores within the hydrogels. After removal from the hexane solution, the hydrogel pieces were weighed^26^. The porosity percentage of the hydrogels was calculated using Eq. 2:

2 $${\text{Porosity }}\left( \% \right){\text{ }} = \left( {\frac{{W_{2} - W_{1} }}{{\rho \;~ \times \;Vs}}} \right)\; \times \;100$$

where W_1_ & W_**2**_ are the weight of hydrogels before and after immersion in n-hexane, ρ is the density of n-hexane (0.665–0.683 g/ml) and Vs is the volume of the hydrogel discs. These experiments were performed in triplicate, and the mean value was calculated.

### Structural features of the Gel-GMA hydrogels and Gel-GMA/GO composites

To confirm the characteristic functional groups present in the synthesized hydrogels and their composites, various analytical techniques were employed. Fourier-transform infrared (FT-IR) analysis was conducted using a Nicolet IS 10 spectrophotometer from Thermo Fisher Scientific, with KBr pellets, covering the wavenumber range from 4000 to 400 cm^− 1^. Proton nuclear magnetic resonance (1 H-NMR) spectroscopy was utilized to verify the structure of the synthesized Gel-GMA hydrogels. For this, 30 mg of lyophilized Gel-GMA was dissolved in 1 ml of dimethyl sulfoxide (DMSO) to obtain a clear solution, and spectra were recorded in DMSO-d6 at 300 MHz on a Varian Mercury VX 300 NMR spectrometer, with trimethylsilane as an internal reference. UV measurements for the GO nano-composite suspension were performed using a Thermo Scientific Evolution 300 UV-Vis spectrophotometer, covering a spectral range of 200–600 nm. Raman spectroscopy was conducted using a Wi Tec 300 R Raman spectrometer with a 532 nm laser line. Structural phase analysis of GO and modified gelatin composites was carried out by recording X-ray diffraction patterns using an XRD Rigaku MiniFlex instrument. Particle diameters of graphite and GO aqueous dispersions were determined using a dynamic light-scattering nanoparticle double-beam analyzer, PSS-NICOMP particle sizer 380ZLS. Morphological analysis of the prepared hydrogels was performed using scanning electron microscopy (SEM). Samples were freeze-dried using a BIOBASE Model Country freeze-dryer under conditions of -43 °C temperature and 0.31 mbar vacuum. For transmission electron microscopy (TEM), a drop of the dispersed solution was placed on carbon-coated copper grids and air-dried at room temperature. Electron micrographs were obtained using a JEOL GEM-1010 transmission electron microscope at 80 kV.

### Evaluation of blood-contacting biomaterials

#### Hemolysis assay

The concentration of free hemoglobin in plasma was assessed by adding cyanmethemoglobin (CMH) reagent, such as Drabkin’s reagent, which rapidly converts free hemoglobin to its cyano-derivative. The absorption of CMH was then measured at 540 nm using a photometer, following the “direct cyanmethemoglobin method”^27^. Hemolysis percentage was calculated using Eq. 3:

3 $${\text{Hemolysis }} = \left( {\frac{{A_{s} - A_{{nc}} }}{{A_{{pc}} - A_{{nc}} }}} \right)\; \times \;100$$

where A_s,_ A_nc_ and A_pc_ are the absorbance values at 540 nm of the supernatant in the sample solution; negative control i.e., normal saline solution (0% hemolysis, ) and positive control i.e., distilled water (100% hemolysis, ), respectively.

#### Coagulation cascade

Prothrombin Time (PT) and activated Partial Thromboplastin Time (aPTT) are widely used laboratory tests to assess extrinsic and intrinsic coagulation pathways, respectively, aiding in the diagnosis of coagulation disorders. To measure blood clotting time, citrated platelet-rich plasma (PRP) was obtained from anticoagulated human whole blood and incubated with the test materials at 37 °C for 30 min. For the PT test, 100 µl of PRP was mixed with 100 µl of PT reagent in a cuvette, placed in the coagulation analyzer, and the time for PRP clotting was recorded. To conduct the aPTT test, 50 µl of PTT reagent (cephalin) was added to 50 µl of PRP for 3 min, followed by the addition of 50 µl of 0.025 M CaCl_2_ solution to initiate clot formation after inactivating the anticoagulant. The time taken for clot formation was then recorded as the activated PTT (aPTT). Shortened clotting times indicate activation of the intrinsic and common pathways of coagulation by the test material^28,29^. These tests were conducted by the private accredited laboratory (El-Esteshary Lab, Maadi, Cairo, Egypt). Our study was conducted in accordance with the relevant Egyptian regulations, the Helsinki Declaration, and the principles of Good Clinical Practice (GCP) and Good Laboratory Practice (GLP), as adopted by our institutional ethics committee: Medical Research Ethics Committee of the National Research Centre.

#### In-vitro degradation

A hydrogel with a predetermined weight was submerged in 20 ml solutions of PBS with a pH of 7.4. One sample was then incubated at 37 °C, and its weight was monitored after 30 and 100 days. The solution was filtered daily, and the weight of the sample was documented. The percentage change in weight was calculated using the formula provided in Eq. 4.

4 $${\text{Weight change }}\left( \% \right){\text{ }} = \left( {\frac{{Initial~Weight - Final~Weight}}{{Initial~Weight}}} \right)\; \times \;100$$

For each solution, three readings were taken, and the mean value was considered as the final % weight change.

#### Cell compatibility

To assess the biocompatibility of materials, cellular viability was evaluated using MTT assay on human lung fibroblast cells (MRC-5 cells, sourced from ATCC, LGC Standards, Poland). For the cytotoxicity assay, cells were seeded in 96-well plates at a concentration of 1 × 10^4^ cells per well in 100 µl of growth medium. After 24 h of seeding, fresh medium containing various concentrations of the test sample was added. Serial two-fold dilutions of the tested material were applied to the cell monolayers in the microtiter plates, and incubation was carried out at 37 °C in a humidified incubator with 5% CO_2_ for 24 h. Control cells were treated without the test sample, with or without DMSO, with the negligible presence of DMSO (maximal 0.1%) having no impact on the experiment. Following incubation, viable cell yield was determined using a colorimetric method.

In brief, after removing the media, each well was treated with 1% crystal violet solution for at least 30 min. After rinsing excess stain with tap water, 30% glacial acetic acid was added to all wells, mixed thoroughly, and the absorbance of the plates was measured at 490 nm using a microplate reader (TECAN, Inc.), after gently shaking. Results were adjusted for background absorbance detected in wells without added stain. Treated samples were compared with cell controls lacking the tested items to determine cytotoxic effects. The optical density was measured with the microplate reader (Sunrise, TECAN, Inc, USA) to ascertain the number of viable cells, and the percentage of viability was calculated using Eq. 5.

5 $${\text{Viability percentage }} = ~\frac{{OD_{t} }}{{OD_{c} }} \times 100$$

where OD_t_ and OD_c_ are the mean optical density of wells treated with the tested sample and that of untreated cells, respectively. The relation between surviving cells and sample concentration was plotted to get the survival curve of each cell line after treatment with the specified sample.

### Statistical analysis

Statistical analysis was carried out employing Microsoft Excel statistical analysis. The standard deviation was calculated for each treatment group (mean ± SD). The level of significance was set to be *p* ≤ 0.05 at confidence level = 95%.

## Results and discussion

### Elucidation of graphene oxide (GO) structure

**Fig. 1:**
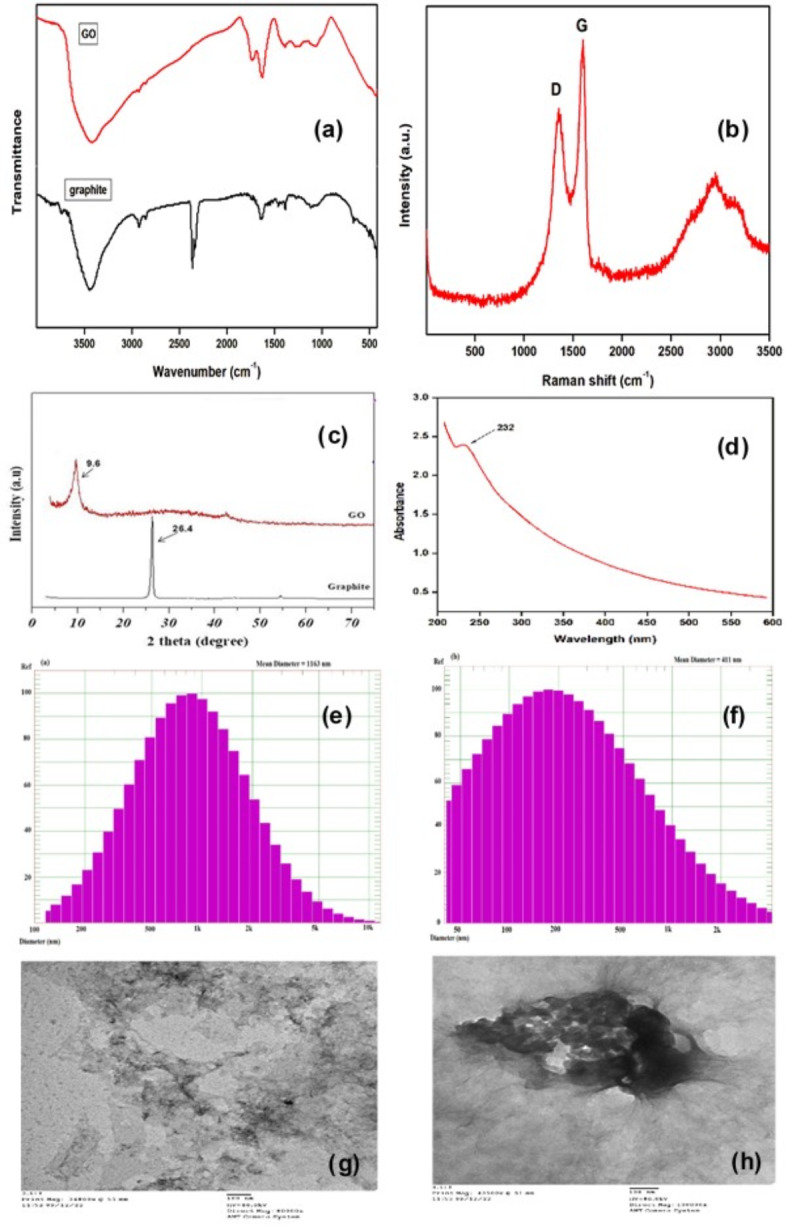
(**a**) FTIR spectra of graphite & GO; (**b**) Raman spectrum of GO, (**c**) XRD patterns of graphite & GO; (**d**) The inset: UV spectrum of GO; (**e**) DLS of aqueous dispersion of graphite; (**f**) DLS of aqueous dispersion of GO and (**f**) TEM images of GO at different magnifications.

Figure 1a displays the FT-IR spectra of graphite and graphene oxide (GO). The synthesized GO derived from graphite flake exhibits distinctive new peaks at specific wavelengths: 3419 cm^− 1^ (O–H stretching), 2920 and 2854 cm^− 1^ (asymmetric and symmetric CH_2_ stretching), 1731 cm^− 1^ (C = O stretching), 1627 cm^− 1^ (C = C stretching), 1384 cm^− 1^ (O–H bending), 1276 cm^− 1^ (C–OH stretching), and 1075 cm^− 1^ (C–O–C stretching). These emerging peaks indicate the presence of various oxygen functional groups in GO, in contrast to graphite, which displays peaks at 1562 and 3434 cm^− 1^, attributed to the skeletal vibrations of the graphene backbone chain and -OH stretching vibrations, respectively^30^. The distance between two layers serves as a significant parameter for assessing the structural characteristics of graphene.

Following the oxidation reaction, the Raman spectrum in Fig. 1b displays the Raman spectra of graphene oxide (GO), providing evidence of modifications to the graphite structure. New bands emerge at 1359 cm^− 1^ (D), 1600 cm^− 1^ (G), and 2715 cm^− 1^ (2D), indicating alterations. Compared to the original graphite, the widths of the D and G peaks are significantly broader^31^, suggesting reduced crystallinity and increased disorder in the graphitic sheets constituting the original material. This disorder arises from the introduction of defects through the incorporation of oxygenated functional groups into the basal layer or a greater increase in oxygenated margins. The heightened intensity of the D band may signify an augmentation in the number of disordered carbon atoms in GO, representing sp^3^ domains, or a substantial reduction in the size of sp^2^ domains within the layer due to oxidation and exfoliation. This implies the coexistence of sp^2^ and sp^3^ hybridization in GO, indicating the presence of both crystalline and amorphous forms of carbon^32,33^. The ratio of D band to G band intensities (ID/IG) reflects the proportion of amorphous and disordered carbon (sp^3^) relative to graphitic carbon (sp^2^), enabling a comparison of the structural order among samples. For graphite, the ID/IG ratio is 0.14, while for graphene oxide (GO), it is 0.81. The higher ID/IG ratio in GO indicates a greater level of structural disorder, suggesting an effective oxidation reaction.

In Fig. 1c, the X-ray diffraction patterns of graphite and graphene oxide (GO) are depicted. A distinctive peak of graphite powder is observed at 2θ = 26.4°, corresponding to the (002) plane^34^. This peak undergoes a shift towards a relatively broad reflection at 2θ = 9.6°, characteristic of the (001) plane, with an inter-layer spacing distance of 9.16 Å between the GO layers. This shift suggests the effective exfoliation and oxidation of graphite powder into GO sheets, as the d-spacing value significantly exceeds that of natural graphite (3.48 Å), indicating a substantial increase in inter-planar distance due to the introduction of oxygenated functional groups. Moreover, the absence of excessive peak broadening in the characteristic graphite peak indicates well-ordered stacking^35^. Figure 1d shows the UV spectrum of GO. The absorbance spectrum of GO reveals a strong absorption peak at 232 nm, attributed to the π-π∗ transition of the C–C conjugated aromatic domains, and a weaker absorption shoulder at 305 nm, due to the -∗ transition of the C = O bond. Similar to the FTIR results, the UV-vis spectrum provides evidence of a significant number of oxygen-containing functional groups, such as hydroxyl, carboxyl, epoxide, and carbonyl, on the GO.

The Dynamic Light Scattering (DLS) technique measures the hydrodynamic radius of dispersed particles by determining their diffusion coefficient and inferring their size using the Stokes-Einstein relation^36^. DLS is a crucial tool for assessing the aqueous behavior of nanoscale graphene materials^37^, as it calculates the hydrodynamic diameter based on the Brownian motion of particles. Figure 1e and f illustrate the average hydrodynamic diameters of graphite and graphene oxide (GO), which are 1163 nm and 411 nm, respectively. The reduction in particle size of the graphite flakes, accompanied by increased polydispersity, suggests the presence of non-exfoliated GO nanosheets. The study indicates that the oxidation of graphite reduces the mean particle diameter to about one-third of its original size, resulting in high polydispersity and positive skewness. Lastly, the morphological features of GO particles were confirmed through TEM analysis, as shown in Fig. 1g,h. The TEM micrographs display flake-like shapes with some degree of agglomeration. Additionally, the GO exhibited a wrinkled surface, indicating loosely bonded layers and a higher interlayer distance, which contributes to its stability and prevents it from collapsing back into a graphitic structure. This increased stability is likely due to the presence of organic functional groups and electrostatic interactions of oxides on the surface, which add extra thickness to the graphene sheets. Furthermore, the rough, wrinkled surface of GO provides increased contact with the matrix, enhancing its integration.

### Formation of modified gelatin composite hydrogel

Hydrogels are widely used in medical applications due to their ability to mimic the extracellular matrix, providing support for tissue regeneration and drug delivery, and their mechanical compatibility with tissues, reducing irritation and immune response. Their soft, hydrated nature enhances biocompatibility, while their elastic properties make them customizable. Additionally, low interfacial tension minimizes protein adsorption and cell adhesion, lowering immune reaction risks. However, their limitations include poor mechanical properties and rapid degradation. These issues can be addressed by adjusting macromer concentration and crosslinking time, though prolonged UV irradiation can reduce cell viability, and increasing reactive double bonds can enhance crosslinking^38,39^.

**Fig. 2:**
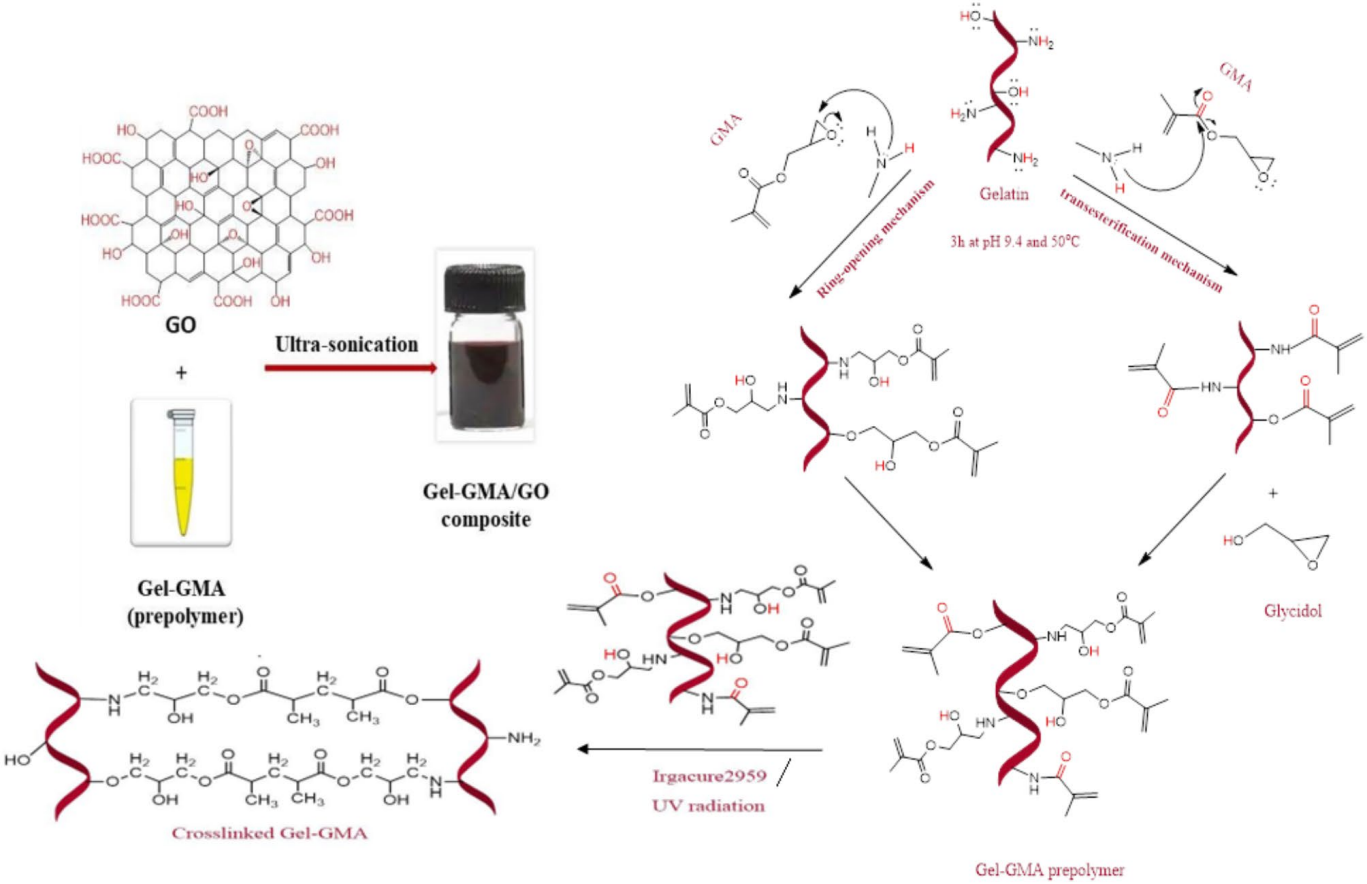
Synthesis of Gel-GMA prepolymer crosslinked Gel-GMA hydrogel and its composite.

Figure 2 illustrates the two possible nucleophilic reaction mechanisms of amine groups on gelatin chains with the methacrylate and epoxide groups of GMA. In one mechanism, the epoxide group of GMA reacts with the amine group via a ring-opening polymerization to form a (3-amino-2-hydroxy) propyl methacrylate group^40^. In the other mechanism, a transesterification reaction occurs, forming a methacryloyl group. The latter mechanism increases pore size as the crosslinker arm length increases, which is crucial for enhancing the swelling of the hydrogels.

The presence of photo-sensitive acrylate groups (CH_2_ = CHCOO−) allows crosslinking to occur under UV irradiation. Irgacure 2959, a photoinitiator with lower cytotoxicity than others, forms soft, elastic, transparent hydrogels with a yellowish tint proportional to the monomer amount. Under 365 nm UV light, Irgacure 2959 dissociates into benzoyl and ketyl free radicals, initiating the crosslinking reactions.

Furthermore, to leverage the biocharacteristics of graphene derivatives and address the fragility and rapid biodegradation of hydrogels, we prepared hydrogel composites by incorporating GO particles. The oxygenated groups on the GO sheets facilitate their dispersion in aqueous media, enhancing the overall properties of the hydrogel composites.

### Structural confirmation of modified gelatin hydrogels and their composites

**Fig. 3:**
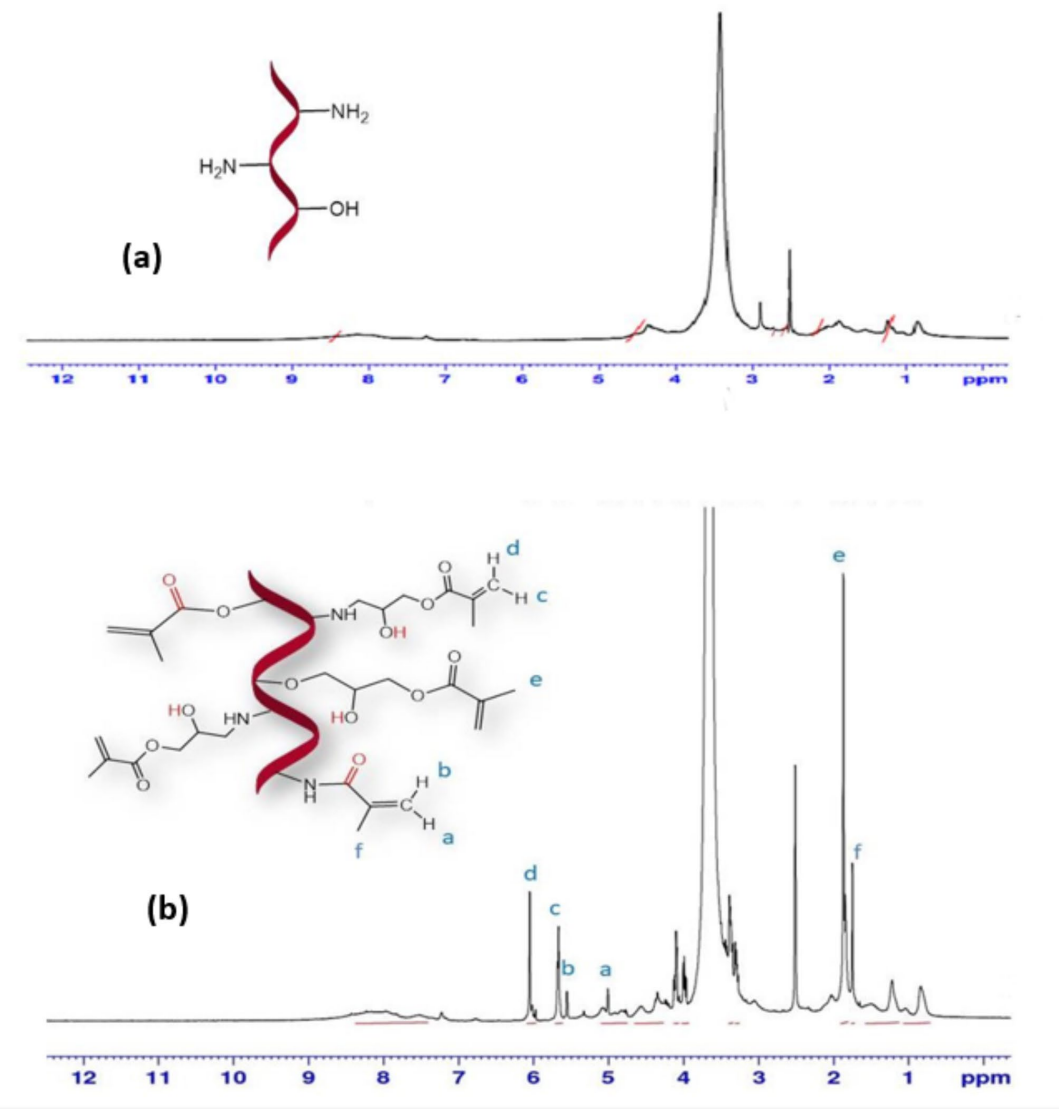
^1^H-NMR spectra of (**a**) Gelatin and (**b**) Gel-GMA prepolymer.

The mechanism of gelatin crosslinking is generally understood to involve the reaction of free non-protonated ε-amino groups (-NH_2_) of lysine or hydroxylysine through a nucleophilic addition-type reaction^41^. Figure 3 shows the ^1^H-NMR spectra of gelatin and Gel-GMA prepolymer. Two signals at 6.1 ppm (d) and 5.7 ppm (c) correspond to the vinyl protons in methacrylate groups. Additionally, the methyl protons of methacrylate groups produce a signal at 1.86 ppm (e), indicating that the modification of gelatin with GMA occurred via a ring-opening polymerization mechanism. Furthermore, the peaks at 5.1 ppm (a) and 5.6 ppm (b) are attributed to the vinyl protons of methacrylate groups from transesterification products, with the methyl protons of methacrylate groups appearing at 1.74 ppm (f). These findings support the possibility of transesterification in the modification reaction.

The results suggest that both mechanisms occur during the modification process. However, the higher intensities of the peaks at 6.1 ppm and 5.7 ppm, associated with the ring-opening mechanism, compared to those at 5.1 ppm and 5.6 ppm, associated with transesterification, indicate that the ring-opening mechanism is more dominant^42^. Additionally, the reaction of GMA with the amine groups of lysine and hydroxylysine residues in gelatin is supported by the decreased intensity of the gelatin band at 2.9 ppm (the methylene protons of lysine). Given the complex composition of gelatin as a mixture of polypeptides, it is challenging to differentiate the resonance peaks between methacrylamide and methacrylate groups via ^1^H-NMR spectra. Therefore, employing a spin-spin decoupling technique in future studies could simplify the NMR spectra analysis.

**Fig. 4:**
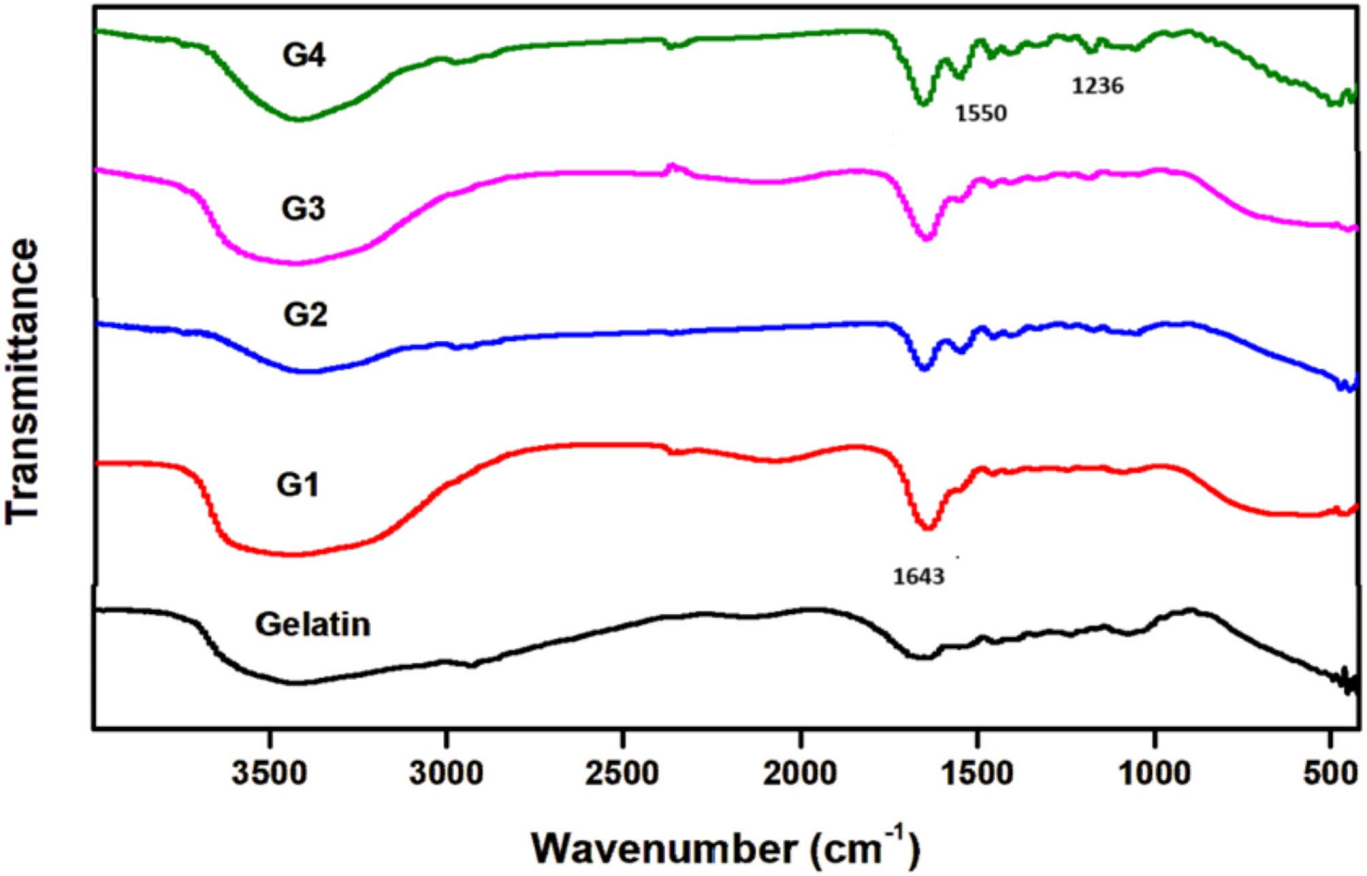
FTIR spectra of gelatin and modified gelatin hydrogels with different GMA concentrations.

Proteins such as gelatin consist of amino acids bonded together by amide bonds. FTIR is a valuable tool for identifying changes in functional groups following chemical modification. Figure 4 depicts FTIR spectra comparing gelatin to modified gelatin hydrogels with varying concentrations of GMA (designated G1 to G4). In pristine gelatin, the C = O stretching vibration at 1643 cm^− 1^ signifies the amide I band, while the amide II band, indicating N–H bending vibration, is evident at 1550 cm^− 1^. Additionally, aliphatic C–H bending vibrations are observed at 1451 cm^− 1^, and the band at 1236 cm^− 1^ denotes C–N stretching and N–H bending in amide III^43^.

The modified gelatin hydrogel derivative displayed characteristic amide bands typical of gelatin protein, including N–H stretching for amide A, C–H stretching for amide B, C = O stretching for amide I, N–H deformation for amide II, and N–H deformation for amide III band. The peak observed at 1235 cm^− 1^, known as amide III, primarily relates to the vibration of the N-H bond (also part of the C-N bond), while the peak at 1545 cm^− 1^, called amide II, is associated with N-H bonds. The amide I peak at 1647 cm^− 1^ indicates the vibration of the C = O bonds. Additionally, peaks at 2979 cm^− 1^ and 3418 cm^− 1^ correspond to amides B and A, respectively. The 2979 cm^− 1^ peak is attributed to C-H bond variations, while the 3418 cm^− 1^ peak reflects the mobility of N-H bonds. It was suggested that, contrary to expectations, the amide bands of methacrylated gelatin shifted to higher frequencies compared to pure gelatin, which contradicts previous findings^44^. Nevertheless, these results affirm the successful synthesis of modified gelatin derivatives^31^.

### Porosity and swelling behaviour of modified gelatin hydrogels

**Fig. 5:**
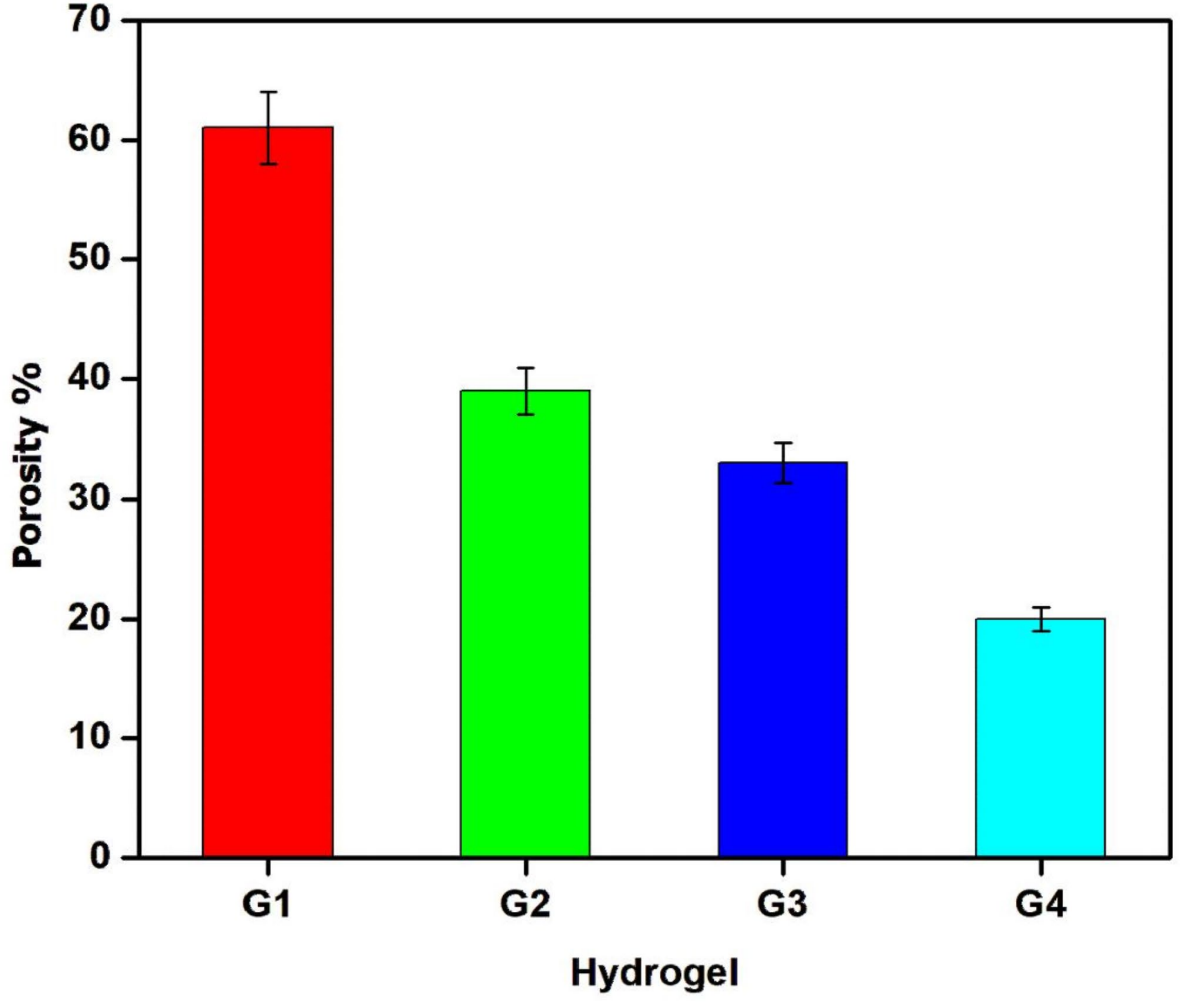
The porosity of lyophilized Gel-GMA hydrogels with different GMA content by n-Hexane.

Porosity and pore architecture profoundly impact the mechanical characteristics of hydrogels, with stiffness typically decreasing as porosity increases. Moreover, biological aspects such as cell viability, proliferation, and migration are significantly influenced by porosity^45^. Therefore, precise control over porosity is crucial as it governs transport properties and affects the behavior of blood-interacting materials. The physicochemical attributes of blood-interacting biomaterials are vital for determining hemocompatibility, essential for both short- and long-term applications. Literature indicates that surface roughness and porosity strongly influence hemocompatibility^46^. Figure 5 depicts the influence of GMA concentration on hydrogel porosity, revealing that higher monomer concentrations result in reduced porosity. This decrease in porosity is attributed to the formation of multiple crosslinks, which tend to diminish the size of interconnected pores and the overall capillary structure.

**Fig. 6:**
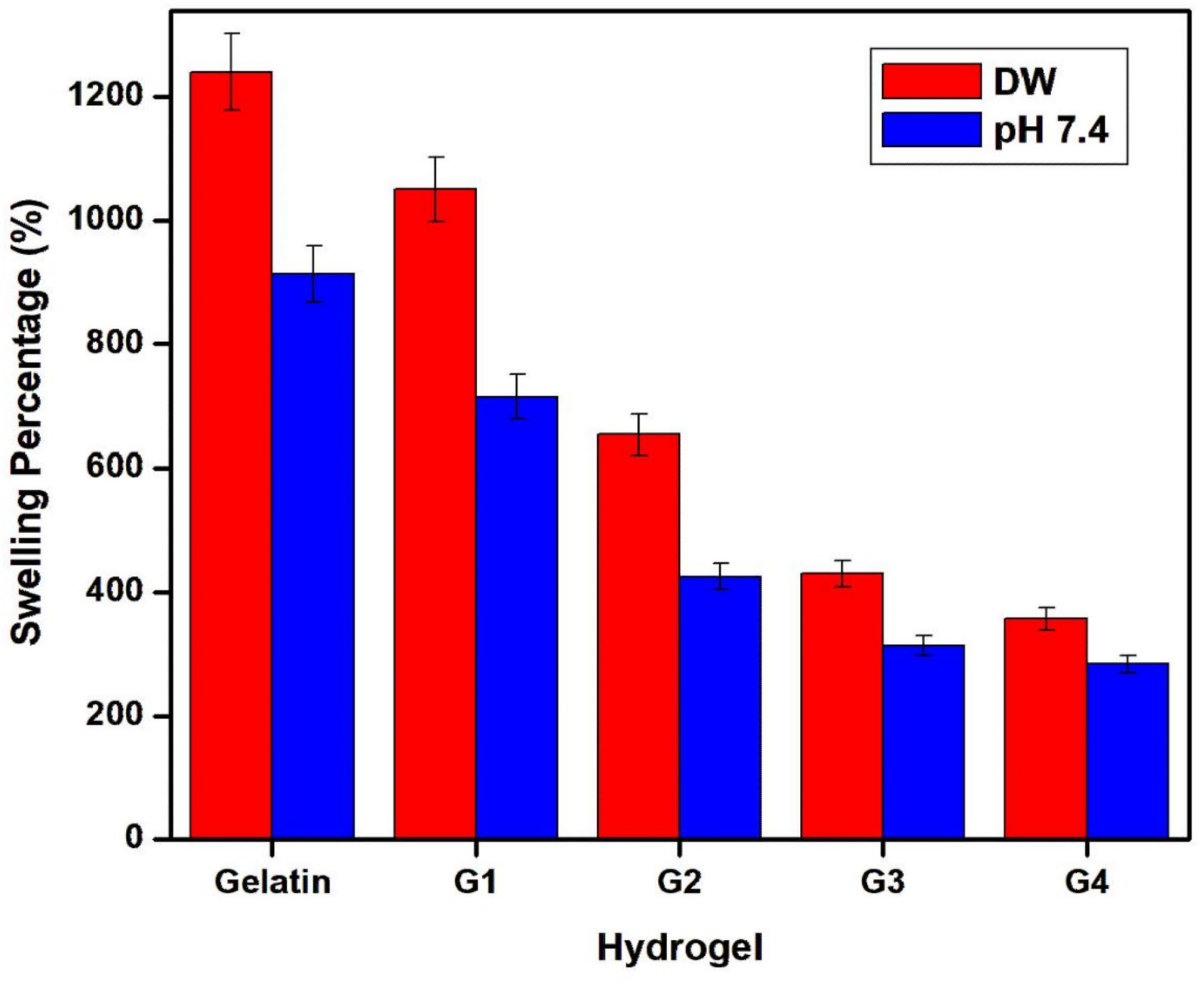
Swelling of Gel-GMA hydrogels with different GMA content in DW and pH 7.4.

The swelling behavior of a network plays a critical role in various applications as it influences solute diffusion, surface properties, mechanical characteristics, and surface mobility. This swelling phenomenon is intricately linked to both the pore size of the polymer network and the interactions between the polymer and the solvent^47^. Hydrogels were subjected to a 24-hour incubation period in distilled water (DW) at room temperature and in PBS at pH 7.4 to reach equilibrium, following which the swelling percentage was calculated and compared (Fig. 6). Maintaining a constant hydrogel percentage, it was observed that the mass swelling increased notably as the degree of methacrylation decreased, indicating a significant influence of methacrylation on the material’s capacity and propensity for absorbing and retaining water within the polymer network.

When a crosslinked hydrogel is immersed in an aqueous solution, water molecules come into contact with the hydrogel surface and infiltrate its polymeric network structure, creating a separation between the non-contacted glassy phase and the contacted rubbery phase^48^. This initiates a moving boundary, causing the rubbery phase network meshes to expand, allowing more water molecules to penetrate the hydrogel, a process known as swelling, where the glassy phase transitions into the rubbery phase. Once swelling equilibrium is reached, the swelling degree stabilizes. Analysis of Fig. 6 reveals that initially, gelatin’s swelling behavior was somewhat diminished by modification. Specifically, “G1” exhibited a swelling percentage of approximately 1050%, highlighting the influence of monomer concentration and resultant porosity. However, further gelatin substitution led to varying degrees of reduced swelling, depending on the substitution percentage. This phenomenon can be attributed to the higher free gel volumes and network voids present in less crosslinked hydrogels^49^, facilitating rapid water uptake via capillary forces rather than slow diffusion. The swelling data are consistent with those obtained from n-Hexane displacement, as discussed earlier. It’s noteworthy that the body maintains blood pH close to 7.4 for optimal metabolic and physiological function. External solution pH values significantly affect hydrogel swelling equilibrium, contingent upon the length of chains between network junctions and the polymer chains’ affinity for solvent molecules.

The observation that hydrogel swelling in water exceeded that in buffer solutions can be attributed to the lower viscosity of water compared to PBS. This lower viscosity allows water to diffuse more readily into the hydrogel matrix than PBS, resulting in less swelling in PBS. Additionally, it’s pertinent to consider osmotic pressure or the balance of chemical potential energy inside and outside the gel network as another factor influencing swelling behavior.

The G2 composite was chosen as a model hydrogel for blood-contact biomaterials based on its relatively high swelling ratio and superior mechanical integrity compared to the other tested samples. These properties are critical for ensuring both functional performance and durability in blood-contact applications, such as vascular catheters.

### Microstructure of modified gelatin hydrogels and their composites

**Fig. 7:**
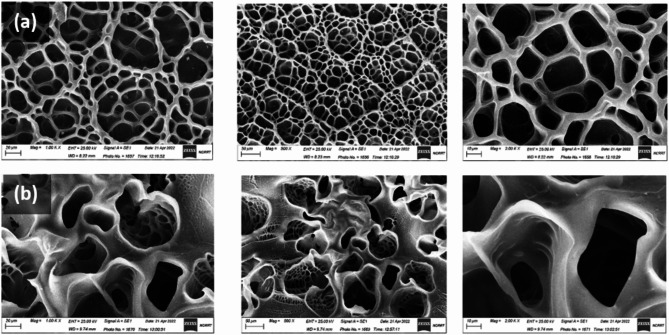
SEM images of lyophilized (**a**) Gel-GMA hydrogels & (**b**) Gel-GMA/GO composites [G2].

Figure 7 illustrates the SEM images of the lyophilized Gel-GMA hydrogels and their composites with graphene oxide (GO), revealing essential morphological features that significantly influence their performance in biomedical applications. The Gel-GMA hydrogels exhibit a well-interconnected porous network with macropores averaging 20 μm in size. This structure is likely due to the extended glycidyl methacrylate (GMA) chains, which promote a high degree of crosslinking, resulting in uniform pore formation. Upon the incorporation of GO, the hydrogel matrix undergoes a remarkable transformation, with the formation of three-dimensional capillary channels extending into the interior. These channels not only enhance the internal connectivity of the hydrogels but also facilitate the absorption of fluids and diffusion processes, which are critical for swelling behavior and other biological functionalities.

The GO’s oxygen-rich functional groups, such as hydroxyl, epoxy, and carboxyl groups, appear to stabilize the porous network by interacting with the polymer chains, ensuring a uniform and well-connected structure without compromising macroporosity. This stabilization enhances the structural integrity of the hydrogels, making them suitable for applications requiring efficient fluid transport, such as vascular catheters. The observed pore sizes exceed the threshold identified in studies by Elbert et al., which emphasized the importance of pores larger than 1 μm for vascularization and nutrient diffusion^50^. The macroporous nature of our hydrogels suggests a significant potential for facilitating cell migration and proliferation, which is further supported by the presence of hydrophilic surfaces that promote cellular interactions. Additionally, similar findings by Chopra et al. confirm that the integration of GO into gelatin matrices enhances pore interconnectivity and mechanical stability while preserving essential swelling characteristics^51^. These structural attributes are particularly advantageous for blood-contacting biomedical devices. The interconnected pores and capillary channels allow for rapid fluid absorption, which improves the swelling capacity and reduces friction during catheter use. Moreover, the high porosity and hydrophilicity minimize platelet adhesion, thereby lowering the risk of thrombus formation and enhancing hemocompatibility. The porous architecture also supports controlled drug release, with the channels serving as pathways for therapeutic agents, making the hydrogels versatile for applications that require both mechanical support and therapeutic delivery. These findings align with research by Xiang et al., who demonstrated the benefits of macroporous hydrogels for improving drainage and toxin absorption in catheter applications^52^.

In conclusion, the Gel-GMA/GO composites present a well-optimized porous structure that aligns with the requirements of advanced biomedical applications. The interconnected capillary networks and stabilized macropores ensure efficient fluid management, cellular interactions, and compatibility with blood-contacting devices. The scientific implications of these findings underscore the potential of these hydrogels to advance the design and functionality of medical devices such as vascular catheters, scaffolds, and drug delivery systems.

### Evaluation of Gel-GMA and composite thereof as blood-contact biomaterials

When evaluating the interactions between medical devices and blood, it’s essential to consider various factors such as device design, materials used, clinical application, environmental conditions during use, and the balance of risks and benefits. Due to the complexity of these factors, it’s challenging to prescribe specific testing methods with detailed requirements. The limitations in our understanding and the precision of available tests for assessing device-blood interactions contribute to this challenge.

#### Hemolysis assay

**Fig. 8:**
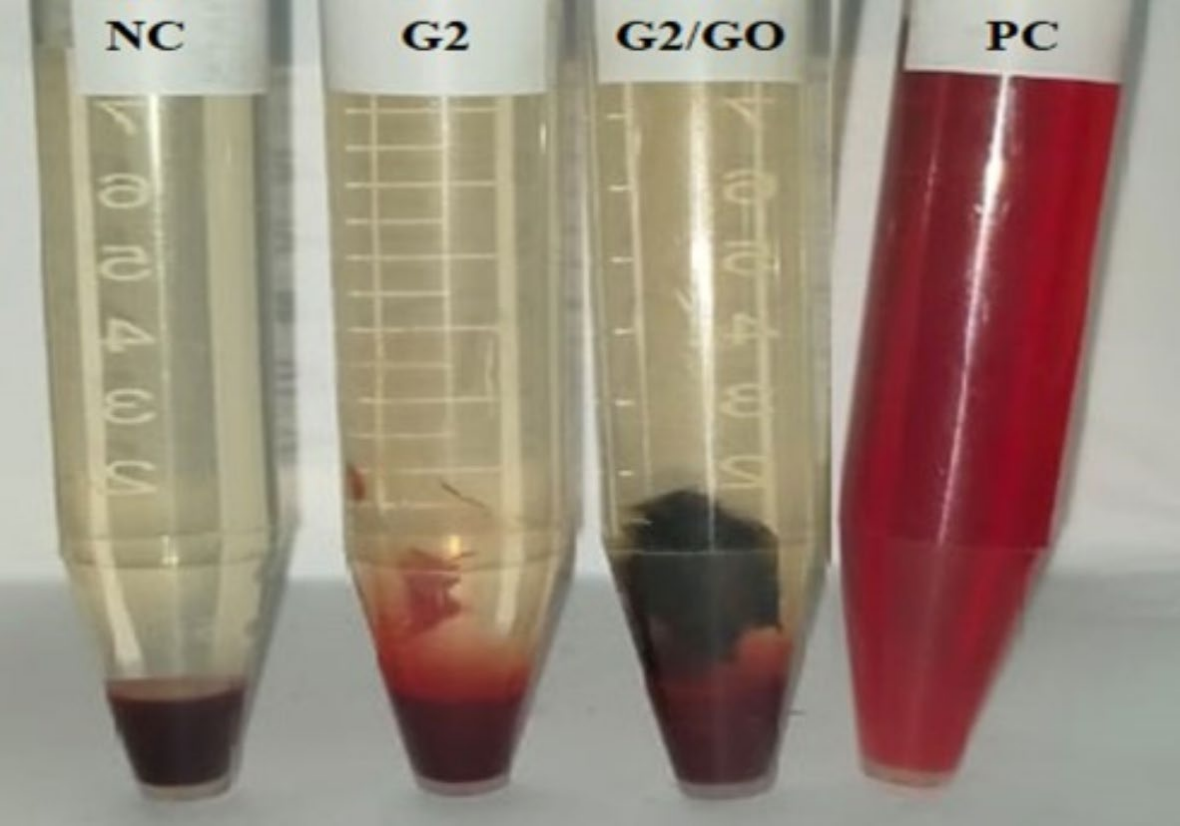
Hemolysis images of fresh human blood samples with saline as a negative control (NC), Gel**-**GMA (G2), (G2)/GO, and distilled water as a positive control (PC).

The development of formulations that are both hemocompatible and low in toxicity presents a significant challenge. Hemolysis, characterized by the release of hemoglobin due to the rupture of red blood cell membranes, is a serious concern associated with blood-contacting medical devices. To assess blood compatibility, the hemolysis assay measures the amount of ruptured red blood cells in contact with a sample, with lower hemolysis rates indicating better compatibility. However, hemolysis can accelerate thrombus formation by promoting platelet adhesion, underscoring its importance as a method for evaluating material compatibility^52^. According to ASTM F-756:17, a hemolysis rate of less than 5% is considered non-toxic^53^. Elevated levels of free hemoglobin in plasma directly indicate red blood cell destruction, which can impair oxygen transport to tissues and organs, potentially leading to tissue toxicity^54^. Figure 8 depicts the hemolysis evaluation of the modified gelatin hydrogels. Remarkably, only the positive control’s supernatant exhibited a red hue, indicating erythrocyte destruction, while the supernatants of the other groups remained transparent, indicating intact erythrocytes. The hemolysis percentages for Gel-GMA (G2) and Gel-GMA/GO hydrogels were 0.54% and 0.50%, respectively, both falling below the 5% threshold indicative of hemocompatibility, similar to saline (negative control). In stark contrast, the hemolysis percentage in distilled water (positive control) was 100%, resulting from red blood cell membrane lysis and hemoglobin leakage. These findings affirm the fascinating blood compatibility of the developed hydrogel/composite. Interestingly, a recent study demonstrated that increasing gelatin concentration in polyvinyl gelatin hydrogel enhances hemocompatibility^55^. Our results align with those obtained for polyurethane/gelatin complex coatings intended for potential vascular applications^56^.

#### Coagulation cascade

Exploring blood clotting on biomaterials crucially informs the development of biomaterials. Conventional biomaterials often lack sufficient blood compatibility, necessitating systemic anticoagulation (e.g., with heparin) during device application. Thus, enhancing the hemocompatibility of bulk materials is imperative, which can be achieved through specific synthesis, material blending, or surface modification to regulate blood response. Thrombus formation on blood-contacting medical devices is intricate. Initially, foreign biomaterials interact with blood, prompting plasma protein adsorption on their surfaces. This adsorption occurs via physical interaction forces like Coulomb and van der Waals forces, hydrogen bonding, and hydrophobic interactions, leading to reversible or irreversible adsorption depending on surface properties. Protein adsorption initiates with smaller, more abundant proteins such as human serum albumin, driven by Fickian diffusion due to its lower molecular weight and high plasma concentration. However, higher molecular weight proteins, despite being less abundant, can displace albumin due to their stronger surface affinity^57^.

Hydrophobic surfaces, prevalent in many synthetic polymers, tend to provoke irreversible protein adsorption. This adsorption layer can stimulate blood coagulation, activate the complement system, and trigger the adhesion and activation of platelets and leukocytes, all of which contribute to undesirable blood-material interactions like thrombosis and inflammation. This phenomenon arises from the displacement of clusters of water molecules from hydrophobic surfaces and protein domains, driven by entropy considerations^2,57^. Conversely, polar and hydrophilic surfaces typically exhibit lower protein adsorption due to repulsive forces induced by a thin, bound water layer on the material surface. Stabilizing this water layer through polar interactions, such as hydrogen bonding, generates a repulsive hydration force that thermodynamically inhibits protein adsorption. Another repulsive force, termed “steric repulsion,” arises from the energetically unfavorable compression of hydrophilic macromolecules immobilized on surfaces. Moreover, the blood coagulation cascade involves a series of proteases that activate each other, culminating in the formation of thrombin and subsequent fibrin polymerization.

Table 1 presents the performance of Gel-GMA and Gel-GMA/GO. Encouragingly, the results of PT and aPTT indicate no significant deviation from normal blood values for either material, suggesting that these hydrogels and their composites do not affect blood chemistry upon contact. This absence of protein adsorption, platelet adhesion, or fibrin development on their surfaces underscores their potential to prevent whole blood clotting, a critical requirement for the long-term efficacy of blood-contacting materials^58^. As noted by Sabino et al., achieving complete blood clotting prevention on biomaterial surfaces remains elusive^59^. Thus, our findings offer promise in this regard. Notably, the gelatin composite exhibits superior blood compatibility and lower prothrombin time compared to the corresponding hydrogel. This enhancement can be attributed to its demonstrated higher porosity, as evidenced by porosity measurement and SEM micrographs, as well as its proven elevated swelling capacity.

**Table 1:** Blood coagulation of Gel-GMA “G2” and its composite.

Test	Control (normal blood)	G2	G2/GO	Normal
PT (Prothrombin time) (sec)	12.5 ± 0.30	14.2 ± 0.40	12.9 ± 0.30	11.0–14.0
aPTT (activated Partial Thromboplastin Time) (sec)	33 ± 0.40	38.1 ± 0.60	34 ± 0.50	25.0–45.0

#### In vitro degradation

Biodegradation refers to the breakdown of a material into simpler components under the influence of biological activity. Alongside biocompatibility, biodegradation stands as a crucial aspect of medical materials^60^. Despite passing cytotoxicity tests, some materials may produce toxic byproducts upon decomposition, rendering them unsuitable for further use. For instance, structures designed for sustained release of active substances must degrade gradually and harmlessly. Conversely, nondegradable implants should not persist in the body, necessitating surgical removal.

Four primary mechanisms govern the decomposition of biodegradable polymers: hydrolysis, oxidation, enzymatic breakdown, and physical processes^61^. Hydrolytic degradation occurs when water in the tissue triggers the degradation of vulnerable bonds in the polymer chain, leading to chain shortening. Oxidative degradation results from the body’s immune response to foreign objects, where immune cells produce peroxides, causing polymer chain rupture and degradation. Enzymatic decomposition involves tissue-specific enzymes facilitating degradation. Physical decomposition, distinct from chemical processes, involves mechanical factors like friction and organism movement, contributing to material breakdown.

Biodegradable hydrogels are intricate three-dimensional structures capable of breaking down into simpler, harmless components that can be eliminated from the body. These hydrogels are crafted using materials susceptible to cleavage through hydrolytic or enzymatic processes^62^. The rate of biodegradation in hydrogels is largely dictated by polymer chemistry. As the biodegradation process unfolds, the structural integrity of the matrix deteriorates, allowing for the release of encapsulated cargo molecules. The unique properties of hydrogels, such as swelling, biocompatibility, and, most importantly, biodegradability, not only govern drug release but also obviate the need for surgical removal after their intended use. In gelatin hydrogel composites, biodegradation primarily occurs through the hydrolysis of amide bonds by water molecules^63^. In the hydrolytic degradation mechanism, water initially interacts with water-labile bonds either directly on the polymer surface or within the polymer matrix, leading to bond hydrolysis. Hydrolysis reactions can be catalyzed by acids, bases, or salts. This hydrolytic scission of polymer chains results in a reduction in molecular weight. As degradation progresses, the molecular weight of degradation products further decreases through additional hydrolysis, allowing them to diffuse from the bulk material to the surface and eventually into the solution, causing significant weight loss. Additionally, the higher the degree of swelling of the hydrogel, the more pronounced the hydrolysis of amide bonds, thereby increasing biodegradability. Studies have indicated that hydrogels with higher degrees of substitution (DS) exhibit slower degradation rates^64^.

Table 2 presents the in vitro degradation profiles of Gel-GMA and Gel-GMA/GO in human blood at pH 7.4 and 37 °C. Our findings are promising as they demonstrate that the prepared nanocomposites can maintain sufficient integrity even in the challenging environment of blood. Specifically, the Gel-GMA/GO hydrogel composite exhibits a degradation rate of only 40.2% after 400 h (approximately 17 days). This slower degradation can be attributed to the robust hydrogen bonding interactions between GO and gelatin functional groups^51^. The presence of these hydrogen bonds ensures that our composite does not undergo rapid or aggressive degradation.

**Table 2:** In-vitro degradation of Gel-GMA and Gel-GMA/GO in PBS (pH 7.4) at 37 °C.

Samples	Weight loss %	
	After 30 days	After 100 days
G2	37 ± 1.4%	65 ± 2.2%
G2/GO	18 ± 1.1%	35 ± 1.9%

#### Cell compatibility

Despite the widespread use of biomaterials in clinical settings, achieving ideal biocompatibility remains a challenge, with potential adverse reactions such as inflammation, fibrosis, infection, and thrombosis being common concerns. The success of implants and their biocompatibility largely hinge on the interactions occurring at the material-tissue interface. By investigating these specific interactions, our understanding of biocompatibility mechanisms can be greatly enhanced, leading to improved biomaterial development.

In endodontic therapy, biocompatibility is paramount due to the direct contact between materials and vital tissues. Cell culture studies serve as a crucial initial step in biocompatibility assessment, offering toxicological insights in a controlled environment that minimizes confounding variables often encountered in vivo. In vitro testing offers advantages in terms of simplicity, cost-effectiveness, reliability, and reproducibility.

MRC-5 human lung fibroblasts were chosen for the cell tests due to their well-established use as a model in biocompatibility studies. These cells are widely recognized for their relevance in evaluating the cytotoxicity and biological performance of biomaterials, including hydrogels, because they represent normal human fibroblast behavior.

Fibroblasts, such as MRC-5, are essential components of connective tissue and are actively involved in extracellular matrix production and tissue remodeling. Their ability to proliferate and interact with biomaterials makes them ideal candidates for assessing cell-material interactions, adhesion, and potential toxic effects. While vascular applications often involve endothelial cells, fibroblasts provide an initial indication of the material’s general biocompatibility and safety for human use.

**Fig. 9:**
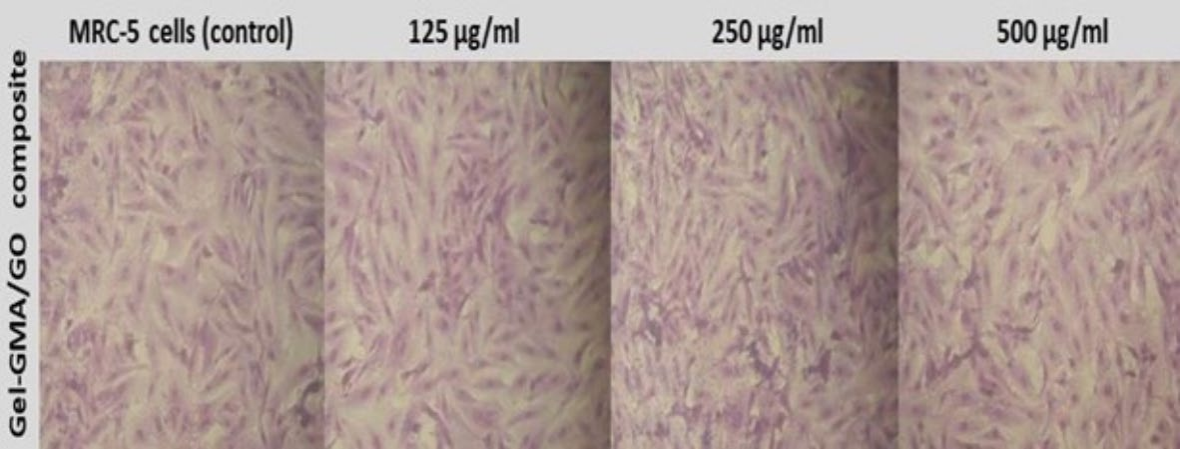
Microscopic images of various concentrations of Gel-GMA/GO composites against MRC-5 cells line (scale bar = 100 μm).

In this study; as shown in Table 3; disk-shaped specimens were prepared, and their extracts were exposed to MRC-5 human lung fibroblasts, a permanent cell line known for providing consistent results compared to primary cells^65^. Compared to untreated cells (blank group), the viability of MRC-5 cells cultured with various concentrations of composite extract remained relatively stable up to 500 µg/ml. However, at 1000 µg/ml, cell viability decreased to 82%. These findings suggest that the Gel-GMA/GO composite [G2] exhibited no cytotoxicity over a wide range of concentrations and even promoted cell growth and proliferation. This conclusion is supported by microscopic images depicting various concentrations of Gel-GMA/GO composites against MRC-5 cell lines, as shown in Fig. 9.

**Table 3:** The cytotoxicity on MRC-5 cells after co-culturing for 48 h with the different concentrations of extracts of Gel-GMA/GO [G2].

Sample conc. (µg/ml)	Viability %	Inhibitory %	S.D. (±)
1000	82.65	17.35	2.71
500	91.74	8.26	1.62
250	96.38	3.62	0.46
125	99.56	0.44	0.32
62.5	100	0	
31.25	100	0	
15.6	100	0	
7.8	100	0	
0	100	0	

In summary, the hydrogel composite developed in this study exhibited promising characteristics, including a hemolysis ratio of less than 5%, a slow degradation rate, and minimal cytotoxicity towards healthy human fibroblast cells. These findings suggest the potential of the composite as a significant advancement in the realm of blood-contacting materials. Furthermore, the presence of graphene oxide (GO) demonstrated notable effects on the assessed biological parameters, underscoring the importance of further investigation into different concentrations of this nano-filler. Additionally, the inclusion of GO may contribute to antibacterial properties and could potentially enhance the mechanical integrity of blood-contacting materials while also modulating biodegradation rates to achieve desired outcomes in terms of hemocompatibility and biocompatibility. Further exploration in this area is warranted to fully understand the potential benefits and optimize the performance of such composite materials.

## Conclusion

Rapid population growth and an aging population are expected to contribute to an increase in disease incidence, particularly impacting the use of blood-contacting materials like catheters, thus imposing a significant economic burden on patients. This study focused on modifying gelatin using glycidyl methacrylate (GMA) to create a photo-curable macromer for subsequent UV crosslinking. Additionally, a composite was prepared by incorporating graphene oxide (GO) into the modified gelatin, introducing various hydrophilic functional groups. Traditional techniques were employed to characterize the structural and morphological features of the hydrogels and composite. NMR studies confirmed that the modification likely occurred via both ring-opening polymerization and transesterification reactions.

The study revealed that increased GMA concentration led to reduced porosity, likely due to the formation of multiple crosslinks that decreased the size of interconnecting pores and capillary structures. Hydrogels exhibited macroporous structures, while the composite displayed interconnected pores, contributing to their high swelling capacity (> 1050%). Hemolysis tests indicated low hemolysis percentages (0.54% for Gel-GMA and 0.50% for Gel-GMA/GO), confirming their hemocompatibility. Prothrombin time tests showed no significant differences compared to normal blood, suggesting minimal blood chemistry impact and no protein adsorption, platelet adhesion, or fibrin development on the material surfaces. In vitro degradation of Gel-GMA and Gel-GMA/GO in simulated blood conditions showed degradation rates of 37% and 18% after 30 days, respectively. Viability tests on MRC-5 cells exposed to composite extract concentrations up to 500 µg/ml remained relatively stable, but viability decreased to 82% at 1000 µg/ml.

Overall, the investigation of key biological features suggests that these hydrogels hold promise as vascular catheter materials. Future efforts will focus on developing a new version of the material capable of controlled drug release, such as anti-infectives, while also addressing thrombus reduction. Additionally, there is interest in exploring novel approaches to develop materials that can prevent the foreign body response altogether.

## Future perspectives

While this study focuses primarily on the evaluation of Gel-GMA/GO hydrogels in terms of their material properties, hemocompatibility, and biocompatibility, several limitations need to be addressed in future research. The current study does not include in vivo assessments, which are crucial for understanding the long-term performance of these hydrogels in actual vascular environments. Additionally, the cytotoxicity testing was limited to MRC-5 cells, and future studies should include assessments using relevant vascular cell types, such as endothelial cells (ECs) and smooth muscle cells (SMCs), to provide a more comprehensive evaluation of the material’s biocompatibility. Furthermore, the mechanical strength, bending resistance, and long-term stability under physiological conditions need to be assessed in more detail.

Moving forward, we aim to address these limitations by conducting in vivo studies to explore the performance of these hydrogels in vascular applications. Moreover, optimization of material properties, such as crosslinking density and the incorporation of other functional materials, will be investigated to enhance the overall performance for catheter and other biomedical applications. These efforts will help us develop a more comprehensive understanding of the material’s potential and guide the future development of Gel-GMA/GO-based hydrogels for vascular and other clinical applications.

## Data Availability

The data that support the findings of this study are available from the corresponding author [H.M. El-Sherif].
